# Expression and prognostic relevance of activated extracellular-regulated kinases (ERK1/2) in breast cancer

**DOI:** 10.1038/sj.bjc.6602655

**Published:** 2005-05-31

**Authors:** K Milde-Langosch, A-M Bamberger, G Rieck, D Grund, G Hemminger, V Müller, T Löning

**Affiliations:** 1 Institute of Gynecopathology, University Clinics Hamburg-Eppendorf (UKE), Martinistr. 52, Hamburg D-20246, Germany; 2 Clinics of Obstetrics and Gynecology, University Clinics Hamburg-Eppendorf (UKE), Hamburg, Germany

**Keywords:** breast cancer, ERK1, ERK2, phosphorylation, prognosis

## Abstract

Extracellular-regulated kinases (ERK1, ERK2) play important roles in the malignant behaviour of breast cancer cells *in vitro*. In our present study, 148 clinical breast cancer samples (120 cases with follow-up data) were studied for the expression of ERK1, ERK2 and their phosphorylated forms p-ERK1 and p-ERK2 by immunoblotting, and p-ERK1/2 expression in corresponding paraffin sections was analysed by immunohistochemistry. The results were correlated with established clinical and histological prognostic parameters, follow-up data and expression of seven cell-cycle regulatory proteins as well as MMP1, MMP9, PAI-1 and AP-1 transcription factors, which had been analysed before. High p-ERK1 expression as determined by immunoblots correlated significantly with a low frequency of recurrences and infrequent fatal outcome (*P*=0.007 and 0.008) and was an independent indicator of long relapse-free and overall survival in multivariate analysis. By immunohistochemistry, strong p-ERK staining in tumour cells was associated with early stages (*P*=0.020), negative nodal status (*P*=0.003) and long recurrence-free survival (*P*=0.017). In contrast, expression of the unphosphorylated kinases ERK1 and ERK2 was not associated with clinical and histological prognostic parameters, except a positive correlation with oestrogen receptor status. Comparison with the expression of formerly analysed cell-cycle- and invasion-associated proteins corroborates our conclusion that activation of ERK1 and ERK2 is not associated with enhanced proliferation and invasion of mammary carcinomas.

Growth factors or cytokines exert positive and negative effects on cell proliferation and differentiation. After binding to their membrane receptors, the message is relayed to the nucleus by a complex system of intracellular signalling pathways. The majority of signalling molecules involved in these pathways are kinases, which are themselves activated by phosphorylation and repressed by their specific phosphatases. The mitogen-activated protein kinase (MAPK) signalling pathway includes a cascade of four groups of kinases (MAPKKKKs, MAPKKKs, MAPKKs and MAPKs). The MAPK kinase kinase kinases of the first level are phosphorylated in response to various extracellular stimuli through interaction with small GTP-binding proteins like Ras, Raf, etc. The activated enzymes then phosphorylate one of 14 kinases of the second level (the MAPKKKs, that is, Raf proteins, MEKK1-4, etc.), which themselves activate one of the MAPK kinases (MEK1 and 2, MKK3–7) of the third level. Finally, these kinases (MAPKKs) activate MAP kinases, which are then able to phosphorylate transcription factors, which regulate the expression of genes involved in cell proliferation or differentiation. Three different, partly interacting signalling pathways have been identified in mammalian cells, leading to the activation of three types of MAP kinases: the MAP kinase JNK (Jun kinase) phosphorylates c-Jun, JunB, ATF2 and ELK1, etc., P38 activates ATF2, ELK-1 and MAX, whereas ERK1 and ERK2 phosphorylate c-Myc, SAP-1, Fra-1 and Fra-2, etc. After phosphorylation, the MAP kinases translocate to the nucleus where they can activate nuclear transcription factors.

The role of the MAP kinases ERK1 and ERK2 has been extensively studied *in vitro*. Since both proteins, although encoded by different genes, show high structural and functional similarity, they are often referred to as ERK1/2. After phosphorylation by the MAPKKs MEK1 or MEK2, the activated ERK proteins (p-ERK1/2) form dimers and are translocated from the cytoplasm to the nucleus (Brunet *et al*, 1999). MEK–ERK activation in breast cancer cell lines occurs after binding of oestrogen to its receptor (Collins and Webb, 1999), stimulation by insulin (Griffiths *et al*, 1998) or binding of growth factors like EGF or PDGF (Whitmarsh and Davis, 1996). In addition, the transformation of mouse mammary epithelial cells by the oncogenes *neu*, Ha-*ras* or *myc* is accompanied by ERK upregulation (Amundadottir and Leder, 1998). Experiments with MEK1 inhibitors have shown that ERK activity correlates with cell proliferation (Seddighzadeh *et al*, 1999) and motility (Krueger *et al*, 2001) in breast cancer cell lines, but does not influence cell invasion. Moreover, the ratio of ERK1/2 to p38 in various tumour cell lines determines whether a cell will proliferate or enter a state of dormancy (Aguirre-Ghiso *et al*, 2003). In a three-dimensional model, treatment of breast cancer cells with MAPK inhibitors leads to a loss of tumorigenic properties (Wang *et al*, 2002), and an intact MAPK pathway is necessary for alveolar morphogenesis in a reconstitution model (Niemann *et al*, 1998).

Transcription factors of the AP-1 family, which include members of the Jun family (c-Jun, JunB, JunD) and Fos family (c-Fos, FosB, Fra-1 and Fra-2) have been identified as targets of MAP kinases (Whitmarsh and Davis, 1996). c-Jun and JunB are phosphorylated by the Jun N-terminal kinase JNK (Mechta-Grigoriou *et al*, 2001). In mouse keratinocytes, ocadaic acid treatment leads to phosphorylation of JunD and FosB by ERK and subsequent activation of an AP-1-responsive promoter (Rosenberger *et al*, 1999). The Fos-related antigens Fra-1 and Fra-2 are phosphorylated by MAPK *in vitro*, which leads to stabilisation, strong conformational change and an increase in DNA-binding affinity (Gruda *et al*, 1994). c-Fos phosphorylation by MAPK leads to its destabilisation and rapid degradation (Tsurumi *et al*, 1995).

In contrast to these *in vitro* data, there are only few and partly contradictory reports on the expression and activity of ERK1/2 in clinical tumour tissues. Therefore, we studied the expression of ERK1 and ERK2 and their phosphorylated forms p-ERK1 and p-ERK2 by Western blot analysis in 148 mammary carcinomas, among them 120 cases with follow-up data. In addition, p-ERK1/2 expression was analysed by immunohistochemistry on paraffin sections from 129 tumours. The results were statistically correlated with clinical and histological data, the steroid hormone receptor status as well as recurrence-free and overall survival. Since 75 cases of our cohort had been characterised before with respect to the expression of the AP-1 proteins, several cell-cycle regulatory proteins (cyclin D1 and E, Rb, Rb2, p16, p21, p27), Ki67 and HER2/neu, the matrix metalloproteinases MMP1 and MMP9 and members of the uPA–PAI-1 system (Bamberger *et al*, 1999; Milde-Langosch *et al*, 2000; Milde-Langosch *et al*, 2004), we analysed possible associations of these factors with ERK1/2 or p-ERK1/2 expression.

## MATERIALS AND METHODS

### Materials

For our studies on clinical tumour tissues, we analysed 148 breast cancer samples of the Hamburg University Hospital, Department of Obstetrics and Gynecology (147 female patients, one male patient; mean age 57 years, range 25–90 years), taken in the years 1991–2000. The only inclusion criteria for this retrospective study were the availability of frozen tissue suitable for protein extraction and (in most cases) follow-up data. Patients treated for primary breast cancer underwent surgery including lumpectomy and dissection of axillary lymph nodes by axillary clearance or modified radical mastectomy. After surgery, the patients received adjuvant treatment (endocrine treatment and/or anthracycline-containing chemotherapy as well as radiotherapy) according to national guidelines.

Histologically, 125 carcinomas were of ductal type, 18 cases were lobular tumours, four cases had mucinous differentiation and one case was a tubular carcinoma. Eight tissue samples were from recurrencies and 140 from primary tumours. Of the primary tumours, 98 were nodal negative and 41 nodal positive and nodal involvement was unknown in one case. By immunohistological oestrogen receptor (ER) assay, 38 cases (26%) were ER negative and 110 tumours (74%) were ER positive. The pathological staging of the primary tumours was carried out as recommended by the UICC. In all, 28 tumours were classified as stage 1, 93 tumours as stage 2 and 18 tumours as stage 3 or 4. Staging data were unavailable in nine cases. According to histological examination, 11 specimens were of low malignancy grade (G1), 75 tumours were classified as G2 and 62 cases were high-grade carcinomas (G3). Follow-up data were available for 120 patients (median follow-up time: 74.2 months, range 6–144 months). A total of 29 patients suffered from recurrence and 21 died from their carcinoma 6–123 months after diagnosis.

After macroscopic examination of the mammary tissue, two adjacent tumour areas were either fixed in 4% buffered formalin for 24 h prior to dehydration and embedding in paraffin or snap frozen in liquid nitrogen and stored at −80°C until further processing. Before freezing, the tissue samples were carefully macroscopically dissected and freed of attached nontumour tissue in order to achieve at least 50% tumour cells.

The mammary carcinoma cell line MCF7, which was used as control on Western blots was cultivated as described (Milde-Langosch *et al*, 2000).

### Immunohistochemistry

For p-ERK1/2 detection, automatic immunostaining on the DAKO Autostainer was performed using the monoclonal antibody phospho-p44/42 MAPK (Thr202/Tyr204), clone E10 (Cell Signaling Technology, Beverly, MA, USA) diluted 1 : 100 and the ChemMate™ Peroxidase/DAB Detection Kit (DakoCytomation, Glostrup, Denmark). This antibody was previously used successfully to visualise phosphorylated ERK1/ERK2 in paraffin-embedded tissue samples (Adeyinka *et al*, 2002). Its specificity was also shown in Western blots (see Results). Serial sections of 4–6 *μ*m were cut from the paraffin blocks and mounted on APES-coated slides, deparaffinised in xylene and rehydrated in graded alcohol to TBS (50 mM Tris, 150 mM NaCl, pH 7.4). Antigen retrieval occurred by microwave treatment for 20 min in 20 mM Tris, 10 mM citrate and 13 mM EDTA, pH 7.8. Blocking of endogenous peroxidase activity and application of the primary antibody were followed by the incubation with biotinylated goat anti-mouse immunoglobulins and streptavidin conjugated to horse-radish peroxidase. DAB (3,3′-diaminobenzidine) chromogen solution and a substrate buffer containing hydrogen peroxide served as substrate system. Tissue sections were counterstained by haematoxylin and permanently mounted. As positive control, a tumour with known expression of the analysed protein in Western blots was used, whereas the primary antibody was omitted for negative controls. The evaluation of immunohistochemistry results was performed independently by two observers. For statistical analysis, the cases were grouped into only three categories according to p-ERK1/2 immunostaining: negative tumours and those showing only weak focal expression (0), carcinomas with 1–20% positive tumour cells (1) and those with more than 20% positivity (2).

The immunohistochemical (IHC) detection of the ER and progesterone receptors (PR) and the oncogene HER2/neu (c-erbB2) was performed in paraffin-embedded samples as described (Bamberger *et al*, 1999; Milde-Langosch *et al*, 2000).

### Western blot analysis

Protein extraction and Western blots were performed as described (Bamberger *et al*, 1999). Briefly, frozen samples were mechanically disrupted to small pieces, and thoroughly homogenised in ice-cold lysis buffer with proteinase inhibitor cocktail (50 mM Tris, pH 6.8, 1% SDS, 10% sucrose, 10 *μ*l ml^−1^ proteinase inhibitor cocktail; Sigma, Taufkirchen, Germany) in a mechanical tissue homogeniser. The homogenate was centrifuged at 13 000 **g** for 5 min, and the protein concentration in the supernatant was determined by standard methods. Equal amounts of protein (20 *μ*g) of each sample were loaded per well. Electrophoresis was performed in a 10% polyacrylamide separating gel with a 3% stacking gel, and proteins were transferred to polyvinylidene difluoride membranes (Immobilon P, Millipore, Eschborn, Germany). After overnight incubation at 4°C in blocking solution (0.1 M maleic acid, pH 7.5, 0.15 M NaCl, 0.005% Thimerosal and 1% blocking reagent; Boehringer Mannheim, Germany), membranes were incubated for 1 h at room temperature with mouse anti-phospho-p44/42 MAPK (clone E10; Cell Signaling Technology, Beverly, MA, USA) diluted 1 : 100 or goat anti-ERK1/ERK2 (C126; Santa Cruz Biotechnology, Heidelberg, Germany) diluted 1 : 4000. As secondary antibody, peroxidase-conjugated anti-mouse-IgG (1 : 2000) or peroxidase-conjugated anti-goat-IgG (1 : 4000; all from Santa Cruz) were used, which were visualised by chemiluminescence reagents (Super Signal West Pico kit, Pierce, Rockfort, IL, USA) with Hyperfilm ECL films (Amersham, Braunschweig, Germany). As control for comparable exposure of chemiluminescent membranes and as standard for densitometry, 20 *μ*g proteins from the cell line MCF7 (for ERK1/2) or a carcinoma with moderate expression (for p-ERK1/2) were always loaded in one well. Band intensities were quantified by densitometry (GS-700 Imaging Densitometer, BioRad, München, Germany). The different protein bands for ERK1 and ERK2 or p-ERK1 and p-ERK2 were measured separately. The intensities of the specific protein bands were calculated as percent intensity of the control sample and corrected for equal actin loading. According to the resulting expression levels, the 148 samples were divided into three groups of 48–50 cases for each protein in order to obtain groups of similar sizes representing tumours with weak, moderate and strong expression.

In all, 75 samples had been analysed before with respect to the expression of the AP-1 proteins c-Jun, JunB, JunD, c-Fos, FosB, Fra-1 and Fra-2. Western blot conditions for all AP-1 proteins have been described in our previous publication (Bamberger *et al*, 1999). Fra-1 and Fra-2 are characterised by two or more bands in Western blots representing different phosphorylation states, which were evaluated separately as described (Milde-Langosch *et al*, 2004). For all AP-1 proteins, the samples were divided into two groups of similar size (weak and strong immunoreactivity) according to their expression levels for statistical correlations.

The expression of the cell-cycle regulatory proteins cyclin D1, cyclin E, Rb, Rb2, p16, p21, p27, the proliferation marker Ki67 and of the invasion-associated proteins MMP1, MMP9 and PAI-1 had also been analysed before by Western blot analysis and densitometry. For statistical analysis, the samples were divided into two or three groups with similar expression levels as described (Milde-Langosch *et al*, 2000, 2004).

### Statistics

The SPSS11.0 program was used for calculation of inter-relationships between the analysed ERK and p-ERK proteins and histological or clinical factors as well as previously analysed proteins by *χ*^2^ test. For the prognostic parameters, the following groups were compared: histological grade G1 and 2 *vs* G3 and 4, stage I and II *vs* IIIand IV, nodal involvement *vs* nodal-negative tumours, ER-negative tumours *vs* ER-positive cases, PR-negative *vs* PR-positive carcinomas, HER2/neu-negative/weak results *vs* moderate/strong HER2neu staining, recurrence-free cases *vs* patients with recurrence during the follow-up period, cases with fatal outcome *vs* patients who were alive at the end of follow-up.

By the same program, Kaplan–Meier analysis of overall and relapse-free survival in groups with different ERK and p-ERK expression was performed. Multivariate Cox's regression analysis was performed using the likelihood ratio test and forward selection of the variables (inclusion limit 10%). Probability values less than 0.05 were regarded as statistically significant. Overall survival was defined between the time interval between the operation and death from breast cancer. Similarly, recurrence-free survival is the time between the operation and the detection of local relapse or distant metastases.

## RESULTS

### p-ERK1/2 immunohistochemistry

Immunohistochemistry for phosphorylated ERK1/ERK2 was performed with 129 mammary carcinomas, two lymph node metastases and eight normal mammary tissue samples. Positive staining was mostly nuclear as expected.

**Figure 1:**
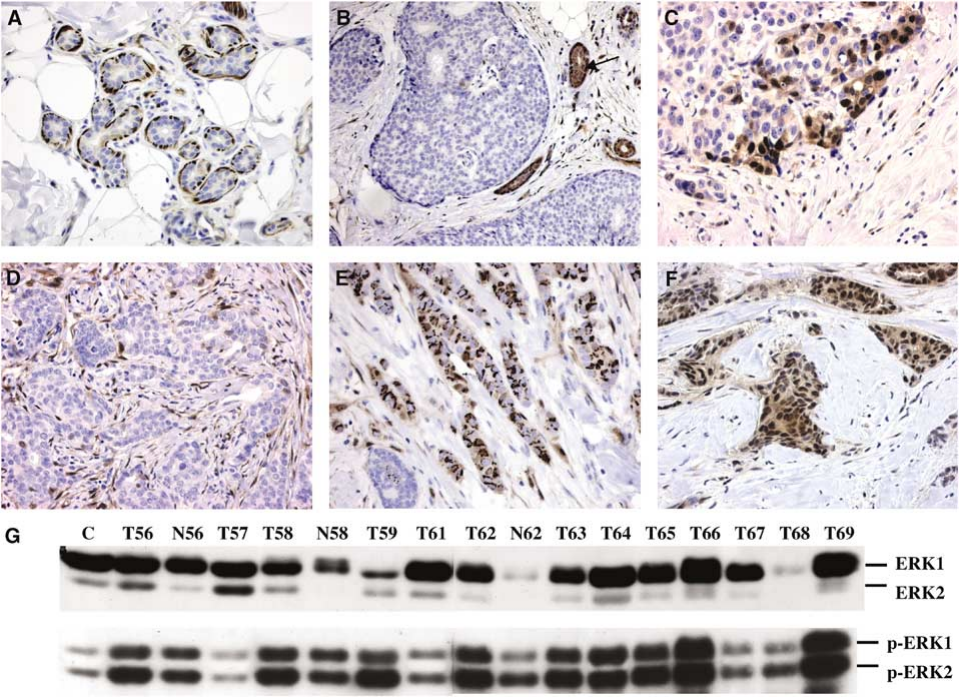
p-ERK1/2 and ERK1/2 detection by immunohistochemistry (**A**–**F**) and Western blots (**G**). (**A**) Positive p-ERK staining in myoepithelial cells around normal terminal lobules in tumour-free mammary tissue from a 65-year-old patient, × 200. (**B**) p-ERK-negative staining result in DCIS areas of a moderately differentiated carcinoma. Strong positivity in ductal epithelia (arrow), × 200. (**C**) Nuclear p-ERK staining in tumour cells at the invasion front of a poorly differentiated ductal carcinoma, × 400. (**D**) p-ERK-negative tumour cells and positive stromal fibroblasts in a moderately differentiated ductal carcinoma, × 400. (**E**) Perinuclear and cytoplasmic p-ERK staining in a poorly differentiated ductal carcinoma, × 400. (**F**) Strong, mainly nuclear immunostaining in tumour cells of a moderately differentiated ductal carcinoma, × 400. (**G**) Western blot analysis of ERK1/ERK2 (p44/p42) and p-ERK1/p-ERK2. T: tumour sample; N: normal mammary tissue sample; C: positive control sample.

Normal ductal or lobular epithelia were either p-ERK1/2-negative or positive in single epithelial cells. Positive immunostaining in normal tissue samples was often found in myoepithelial cells surrounding the epithelia and in smooth muscle cell bundles, and was detected less frequently in stromal fibroblasts (Figure 1A). In carcinoma tissue samples, normal ductal structures adjacent to the tumour frequently showed cytoplasmic and nuclear p-ERK staining (Figure 1B)

Cells of ductal carcinoma *in situ* (DCIS) areas in the neighbourhood of invasive tumours were either negative (Figure 1B) or positive in the outer cell layer of the cell mass.

Invasive carcinomas were often characterised by a striking heterogeneity showing areas of strong nuclear immunoreactivity in tumour cells next to p-ERK1/2-negative regions. Generally, solid tumour masses were mostly negative or weakly stained, whereas p-ERK1/2 positivity was often observed near the invasion front (Figure 1C) or in small tumour cell groups. In Figure 1, examples of tumours with strong (Figure 1F) and absent immunostaining (Figure 1D) are shown. Stromal fibroblasts exhibited different degrees of p-ERK positivity and often displayed strong immunostaining independent of the pERK1/2 expression in tumour cells (Figure 1D). In contrast to the mostly nuclear staining in most cases, strong cytoplasmic reactivity was detected in one tumour (Figure 1E). Both lymph node metastases analysed in this study were negative for p-ERK1/2. There was no detectable difference in p-ERK immunoreactivity between paraffin sections from older blocks (from 1991 to 1994) *vs* newer ones, which indicates that the phosphoepitopes remained intact in paraffin-embedded tissue during long time spans.

For statistical analysis, the cases were divided into three groups: p-ERK-negative or focally positive tumours (*n*=39), tumours with positive pERK staining in less than 20% (*n*=43) and carcinomas with more than 20% positive tumour cells (*n*=47). Stronger p-ERK1/2 immunostaining in tumour cells was significantly associated with early-stage (*P*=0.020) and negative nodal status (*P*=0.004; Table 1). There were no associations of p-ERK1/2 immunoreactivity with age, histological type and grading, ER and PR status, as well as with Ki67 and HER2/neu expression (not shown).

### p-ERK1 and p-ERK2 Western blot results

For immunoblots, the same antibodies used for immunohistochemistry were used, resulting in two bands of 42 and 44 kDa (Figure 1G). Densitometry and statistical analysis were performed separately for both kinases. As positive control, a carcinoma sample showing moderate p-ERK expression was chosen. Relatively to this control, the mean p-ERK1 expression in the carcinomas was 125.9% (range 1–683%), whereas the mean expression of p-ERK2 was 85.8% (range 1–587%).

In the normal tissue samples (*n*=11), high variations of p-ERK1/2 expression were observed. The mean expression levels in this group were 174% for p-ERK1 (range 1–863%) and 126.5% for p-ERK2 (range 1–344%). In pairs of tumour and adjacent normal tissues, p-ERK1/2 expression was either higher (*n*=2), weaker (*n*=2) or nearly identical (*n*=2) in the nontumour samples.

For statistical analysis, the carcinomas were divided into three groups of similar size (*n*=48–50 cases) according to their expression of p-ERK1 and, separately, p-ERK2. There were significant correlations of both phosphorylated kinases with each other (*P*<0.001). Although the extracts analysed by Western blots never represent proteins from tumour cells only, but always also include proteins from stromal fibroblasts and, partly, myoepithelial or ductal epithelial cells which might also express activated kinases, we found correlations of p-ERK1/2 expression in Western blots with IHC p-ERK1/2 expression in tumour cells (*P*=0.030 and 0.048 for p-ERK1 and p-ERK2, respectively; not shown). This indicates that most of the activated ERK1 and ERK2 in the protein extracts used for immunoblots are from tumour cells.

By *χ*^2^ test, we found a significant inverse correlation of p-ERK2 expression with tumour stage (*P*=0.014; Table 1). No significant correlations with age, histological type and grade, nodal involvement, ER and PR status, Ki67 expression and HER2/neu positivity were found.

### ERK1 and ERK2 Western blot results

The mouse antibody used in this study reacts with ERK1 (p44) and, to a lesser extent, with ERK2 (p42). Both proteins were analysed on Western blots and quantified separately by densitometry (Figure 1G). In comparison with the mammary carcinoma cell line MCF7, which served as positive control and was set as 100%, the mean expression levels in the carcinomas were 183% for ERK1 (range 1–863%) and 80% for ERK2 (range 1–314%).

In the normal mammary tissue samples (*n*=7), mean expression levels of 57.3% for ERK1 (range 3–99%) and 42.0% for ERK2 (range 5–93%) were observed. In pairs of tumour and normal tissue, ERK1 expression was higher in the tumour in five of six cases, and ERK2 expression was higher in all carcinomas.

Statistical analysis was performed as described for p-ERK1/2. There was a highly significant correlation of ERK1 with ERK2 protein levels (*P*<0.001). In addition, significant associations were found for ERK1 with p-ERK1 (*P*<0.001) and p-ERK2 (*P*=0.001) as well as for ERK2 with p-ERK2 (*P*=0.014). With patient's age, histological type, grading, stage, PR status, Ki67 and HER2/neu expression and nodal status, no significant associations were found (not shown). For the ER status, a positive correlation with ERK1 expression was observed (Table 1).

### Correlation of ERK1/2 and p-ERK1/2 expression with follow-up data

**Figure 2:**
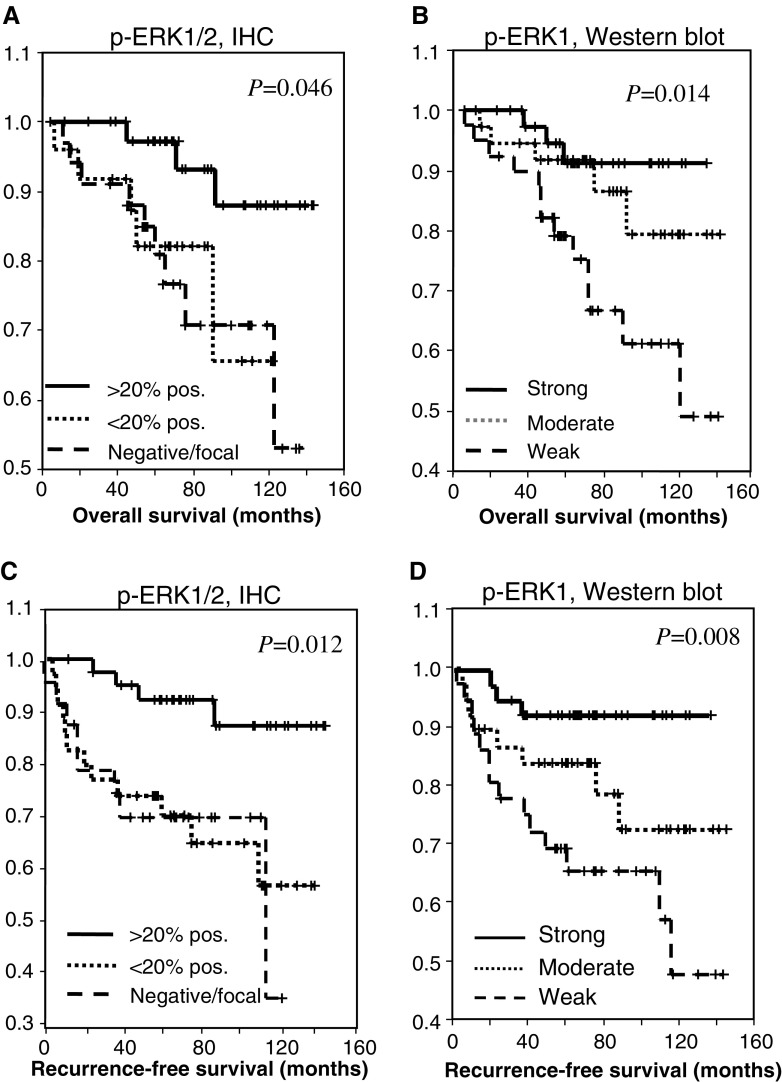
Kaplan–Meier plots of overall survival (**A** and **B**) and recurrence-free survival (**C** and **D**) in groups defined by p-ERK1/2 immunohistochemistry (**A** and **C**) and p-ERK1 Western blots and densitometry (**B** and **D**). The cases were grouped as described in the Materials and Methods.

During the follow-up period, 29 patients suffered from a recurrence and 21 patients died from their disease. By *χ*^2^ test, we found significantly higher numbers of recurrences in cases with negative/focal p-ERK1/2 staining by immunohistochemistry and significantly more frequent death of disease (DOD) or relapse in patients with weak p-ERK1 expression in Western blots. For p-ERK2 expression, only a trend pointing to an inverse correlation with the number of relapses or death was observed (Table 1). Kaplan–Meier analysis showed a significantly longer recurrence-free and overall survival in patients with high p-ERK1/2 expression (>20% tumour cells) as determined by immunohistochemistry and in cases with strong p-ERK1 expression as measured by Western blots and densitometry (Figure 2). For p-ERK2, ERK1 and ERK2, the differences in overall and relapse-free survival were not significant (not shown).

In order to analyse if the expression of phosphorylated ERK proteins is an independent prognostic indicator, a multivariate analysis also including the variables stage, grading, ER status and nodal involvement was performed. By Cox's regression analysis of the Western blot results for p-ERK, high-stage and low p-ERK1 expression were independent predictors of early recurrence, whereas poor differentiation (G3) and low p-ERK1 expression were significant predictors of short overall survival (Table 2). Regarding the IHC results, negative/weak p-ERK expression was the only independent prognostic parameter for early relapse, whereas high grading and negative/weak p-ERK immunoreactivity were independent predictors of DOD (Table 2).

### Correlations with the expression of cell-cycle regulatory proteins and Ki67

The expression of the cell-cycle promoting cyclins D1 and E, the cell-cycle inhibitors Rb, Rb2, p16, p21 and p27, and the proliferation marker Ki67 had been studied before by Western blot analysis or, for Ki67, by immunohistochemistry in 75 tumours from our cohort (Milde-Langosch *et al*, 2000). In order to analyse if there are correlations with the expression levels of ERK1/2 or p-ERK1/2, statistical analysis by *χ*^2^ tests was performed.

There were significant associations of ERK1 expression with cyclin D1 protein levels and of ERK2 expression with cyclin D1, p16 and Rb immunoreactivity. In addition, weak, nonsignificant associations (*P*>0.05) of ERK2 and Rb2 expression and of high p-ERK1/2 and low cyclin D1 levels were found (Table 1). No correlations of ERK and p-ERK levels with p27, p21, cyclin E or Ki67 expression were detected.

### Correlations with the expression of invasion-associated proteins

In a prior study, we examined the expression of the matrix metalloproteinases MMP9 (gelatinase B) and MMP1 (collagenase 1) and the inhibitor of the urokinase plasminogen-activator (PAI-1) in patients of the same cohort (Milde-Langosch *et al*, 2004). For MMP1, protein levels of the proenzyme and the cleaved, active enzyme were analysed separately by Western blots and densitometry.

By statistical evaluation, we found significant correlations of high p-ERK1 protein levels with low MMP9 and high MMP1 (active form) expression. In addition, high ERK1 expression was associated with stronger PAI-1 immunoreactivity. In contrast, ERK2 and p-ERK2 expression and p-ERK1/2 IHC staining were not associated with any of the analysed invasion-associated factors.

### Correlations with the expression of AP-1 transcription factors

The expression of the Jun proteins c-Jun, JunB and JunD as well as the Fos family members c-Fos, FosB, Fra-1 and Fra-2 in the analysed mammary tumour tissues was described in our prior publication (Bamberger *et al*, 1999). Since some of these transcription factors are phosphorylated by activated ERK proteins leading to an increased DNA-binding and transactivating capacity, we compared the expression levels of the AP-1 proteins with those obtained for ERK1/2 and p-ERK/2 in the present study.

For the phosphorylated, active kinases, no significant correlations with AP-1 transcription factors were found. High p-ERK1 expression as found in Western blots was only weakly associated with high FosB and low JunD levels (Table 1). In contrast, the unphosphorylated ERK1 showed a significant positive correlation with FosB and Fra-2 expression, and ERK2 was associated with JunD, c-Fos and Fra-2. No significant correlation with the phosphorylated forms of Fra-1 and Fra-2 was found, except a weak trend for a higher phosphorylated Fra-1 band intensity in tumours with absent/weak p-ERK1 expression (Table 1).

## DISCUSSION

Experimental data with breast cancer cell lines have shown that MAP kinases are involved in normal alveolar morphogenesis (Wang *et al*, 2002), and that high ERK activity correlates with proliferation and motility of breast cancer cells (Seddighzadeh *et al*, 1999). In contrast, there are only few studies dealing with ERK1/2 expression and activation in human clinical tumour tissues.

Investigations on nonmammary tumour types gave partly controversial results regarding the prognostic significance of ERK expression: in salivary gland mucoepidermoid carcinomas, high levels of phosphorylated ERK1/2 in tumour cells were associated with early progression (Handra-Luca *et al*, 2003). In contrast, ovarian tumour patients with high ERK1/2 and p-ERK1/2 expression in pleural effusions had a better overall survival than women with low ERK or p-ERK values (Givant-Horwitz *et al*, 2003). Interestingly, ERK expression and activity in these cases was increased after chemotherapy. In prostate cancer, p-ERK1/2 staining intensity in tumour cells declined with disease progression (Malik *et al*, 2002), and in small cell lung carcinomas, tumours with cytoplasmic expression of p-ERK had a better prognosis than p-ERK-negative cases, whereas no prognostic value was found for nuclear immunoreactivity (Blackhall *et al*, 2003).

With respect to breast tumours, earlier studies suggest an involvement of activated MAP kinases in carcinogenesis and progression. Sivaraman *et al* (1997) reported an increased enzymatic MAPK activity in cytosols from 12 mammary carcinomas as compared to normal tissues or benign lesions, and Salh *et al* (1999) found increased ERK1 and ERK2 amounts after immunoprecipitation relative to normal tissue samples, although ERK2 staining intensity in immunohistochemistry was actually reduced in tumour cells. These results can be partly explained by the lower percentage of epithelial cells in the normal tissue samples. Our IHC and Western blot data point to high variations between the tumours with a downregulation of p-ERK1/2 expression in many carcinomas and overexpression of the activated kinases in other tumours.

In another study, MAPK activity in cytosols from 131 mammary carcinomas was assayed by an enzymatic test and found to correlate positively with nodal involvement, and with a higher risk of relapse, although these associations were not significant in uni- and multivariate analysis (Mueller *et al*, 2000). This is in contrast to our results where high expression of activated ERK1/2 is significantly associated with a long recurrence-free survival. One explanation for this apparent discrepancy might be the different processing of the breast cancer samples: for the biochemical test, the specimens were pulverised in the presence of a mild lysis buffer followed by ultracentrifugation, and only the supernatant was used for MAP kinase measurements. In contrast, we used a buffer containing the strong detergent SDS for protein extraction from tissue samples in order to solubilise as many proteins as possible. In our experience, mild lysis buffers are not suitable for assaying nuclear proteins, since cell nuclei remain intact and are then sedimented by high-speed centrifugation. As members of the intracellular signalling pathways, the unphosphorylated MAP kinases are localised in the cytoplasm. After phosphorylation, they translocate to the nucleus where they can activate various transcription factors (Brunet *et al*, 1999). Therefore, an enzymatic test for MAP kinase activity in cytosolic extracts probably measures only a fraction of the total p-ERK activity.

From a technical view, our present investigation is more comparable to two IHC studies, which both conclude that activated ERK1/2 might be involved in tumour progression and a worse prognosis of breast cancer patients. Both studies differ in the antibodies used: in the first (Adeyinka *et al*, 2002), a polyclonal phospho-p44/p42 (Thr 202/Tyr 204) antibody (New England Biolabs, Frankfurt a.m., Germany) was employed, whereas in the second (Gee *et al*, 2001), a polyclonal anti-ACTIVE™ MAPK antibody (Promega, Mannheim, Germany) for dually phosphorylated ERK1/2 forms was used. In our study, we used a monoclonal phospho-p44/p42 (Thr 202/Tyr 204) antibody (Cell Signaling Technology).

Yet, variations in the used antibodies are probably not the main reason for different results. In the IHC study of Adeyinka *et al* (2002), follow-up data were presented for only 29 ER-positive, nodal-negative patients, which were treated with tamoxifen. No significant differences in response to therapy were observed between these groups, indicating that activated MAPK is not a marker of endocrine sensitivity. Comparing the p-MAPK positivity rates of 90% in a group of nodal-positive tumours (*n*=21) and 48% in a group of mixed nodal status, the authors conclude that activated MAPK might be a marker of a poor prognosis. This assumption was not confirmed by statistical methods like uni- or multivariate analysis. In another study (Gee *et al*, 2001), 90 carcinomas were analysed by IHC with a positivity rate of 72% and an intratumour heterogeneity similar to our observations. High IHC p-ERK1/2 reactivity in these cases was associated with poor response to antihormonal therapy and shorter survival in ER-positive cases and in all patients (Gee *et al*, 2001). In addition, multivariate analysis revealed a significant correlation of short survival and positive p-MAPK immunostaining in the subgroup of ER-positive cases. In contrast to our cohort, all tumours in this study were either locally advanced or metastatic and treated with antihormonal therapy, and 82% of the patients died during follow-up (in contrast to 17.5% in our group). Therefore, a comparison of both studies is difficult and additional investigations will be needed to clarify the discrepancies.

In contrast to these studies, our present data have shown that high p-ERK1 expression in breast cancer tissues is an independent and significant predictor of a favourable prognosis. The correlation of negative p-ERK staining results with shorter relapse-free survival was independently found using two technical approaches – the IHC p-ERK1/2 detection and Western blot analysis – which corroborates this conclusion. Although for the other kinase, p-ERK2, only a weak, nonsignificant correlation with relapse or survival was found, the association of high p-ERK2 expression with early stage does not point to a role of this kinase in tumour progression.

The major advantage of Western blot analysis with p-ERK1/2 antibodies is the possibility to differentiate between both activated kinases (p-ERK1 and p-ERK2), whereas its major drawback is the failure to discriminate between protein expression in tumour cells and accompanying nonmalignant cell types. By immunohistochemistry, p-ERK1/2 protein expression in tumour cells only was calculated. By the parallel use of both methods and the correlations of the results, we could confirm that the p-ERK proteins detected by Western blots are mostly derived from tumour cells. In addition, our data suggest that the correlations of the immunohistochemically detected p-ERK1/2 levels with recurrence-free survival are mainly due to p-ERK1 expression since no statistically significant correlations were found for p-ERK2 (Table 1).

Interestingly, high p-ERK1 protein levels also show correlations with low MMP9 and strong MMP1 expression. Similar to ERK1/2, both MMPs and PAI-1 are expressed by tumour cells and stromal cells in varying proportions (Brummer *et al*, 1999; Dublin *et al*, 2000). In a prior study with the same cohort, expression of the active MMP1 enzyme was associated with ER positivity and a negative nodal status, whereas high MMP9 protein levels correlated with nodal involvement (Milde-Langosch *et al*, 2004). Since MMP9 is well known as a negative prognostic indicator (Kupferman *et al*, 2000), the association of low MMP9 with high p-ERK1 levels fits to our result that high ERK activity is associated with long recurrence-free survival times in breast cancer, although it is no proof of a causal relationship between both factors.

As for the analysed cell-cycle regulatory proteins and Ki67, there were no significant associations with p-ERK1 or p-ERK2 expression, which indicates that there is no direct involvement of these activated kinases in breast cancer proliferation. In immunoblots, all of the cell-cycle regulators are only weakly expressed in normal mammary tissue samples relative to most carcinomas (Milde-Langosch *et al*, 2000). Therefore, the results are probably not due to nontumour cells within the analysed tissue samples. Interestingly, experiments with the breast cancer cell line MCF7 have shown that inhibition of proliferation by TPA is accompanied by high ERK2 expression (Alblas *et al*, 1998). This is in agreement with our results, which are in favour of a growth-inhibiting role of ERK1/2 in breast cancer cells.

The results obtained with the unphosphorylated kinases ERK1 and ERK2 differ from those obtained for the activated enzymes. We found no significant correlations of these proteins with classical prognostic and histological parameters and with recurrence-free or overall survival. Instead, high expression of both enzymes was associated with overexpression of cyclin D1, and ERK2 levels correlated with Rb and p16 expression. Since cyclin D1 is associated with a favourable prognosis in breast cancer (Barnes and Gillett, 1998) and Rb and p16 are well-known tumour-suppressor genes, these results underline that ERK1 and ERK2 are not involved in proliferation in mammary carcinomas. Yet, the association of ERK1 with PAI-1 expression indicates that ERK1 might be involved in the regulation of invasion of tumour cells.

An additional aspect of this study was the comparison of ERK1/2 proteins and their downstream effectors, the AP-1 transcription factors. Similar to most cell-cycle proteins, the expression of c-Fos, Fra-1, Fra-2 and, to a lesser degree, FosB is absent or weak in normal mammary tissue samples, and the data obtained from Western blots with breast cancer specimens probably reflect their expression in tumour cells (Bamberger *et al*, 1999). Since Gruda *et al* (1994) reported a stabilisation of Fra-1 and Fra-2 after phosphorylation by MAP kinases, we expected correlations of these Fos-related proteins or their phosphorylated forms with activated ERKs (p-ERK1 and -2). Yet, we only found weak, nonsignificant associations of strong p-ERK1 expression with high FosB and low JunD and p-Fra-1 protein levels. Fra-1 and Fra-2 are phosphorylated not only by MAP kinases but also by PKA, PKC and cdc2 (Gruda *et al*, 1994). In contrast to the other kinases, MAPK induce a conformational change, which results in a strikingly reduced mobility (Gruda *et al*, 1994). The position of the p-Fra-1 band observed in our tumour samples (Bamberger *et al*, 1999) indicates that the protein was not phosphorylated by ERK1/2, but probably by another kinase. Thus, phosphorylation of Fra-1 and Fra-2 by activated ERK1/2 obviously does not play a major role in the regulation of AP-1 activity in mammary carcinomas *in vivo*.

Our own prior investigations have shown that FosB is highly expressed in more differentiated, hormone-receptor-positive tumours (Milde-Langosch *et al*, 2003). Thus, the comparison of p-ERK expression with AP-1 protein results confirms our conclusion that overexpression of these activated MAP kinases is associated with a better prognosis. The correlations found for the nonphosphorylated ERK proteins with c-Fos and Fra-2 expression (Table 1) might not reflect an effect of MAP kinases on AP-1 proteins, since these associations were not found with the activated enzymes, but rather point to a regulation of ERK expression by AP-1 or common regulatory mechanisms.

Knowing the *in vitro* data that have shown an involvement of ERK1/2 activation in proliferation and motility of breast cancer cells, our experiments with clinical tumour tissues have surprisingly shown opposite results since high levels of the activated kinases were associated with a better prognosis. MAP kinases are members of the signalling pathway, which transduces signals from membrane receptors to the transcription machinery in the nucleus. During tumour progression, the cells often become more and more independent of these extracellular stimuli. In breast cancer, tumour growth is stimulated by oestrogens during tumorigenesis, but highly malignant and progressive tumours are often characterised by a loss of ERs and hormone-independent growth. Similarly, the phosphorylation of target genes by activated ERK1 or ERK2 might be dispensable in advanced mammary carcinomas. Therefore, attempts to target the MEK-ERK pathway for therapy of breast cancer might not be successful in such cases (Shen and Brown, 2003). Additional investigations will be necessary to analyse the underlying mechanisms leading to strong proliferation and invasion in these tumours. Furthermore, the relevance of phosphorylated ERK proteins as prognostic or predictive indicators in breast cancer should be analysed in future larger studies.

## Figures and Tables

**Table 1:** Correlations of ERK1, ERK2, p-ERK1and p-ERK2 expression with histological and clinical tumour parameters, cell-cycle regulatory and invasion-associated proteins and AP-1 transcription factors

**Correlation with: (detection method)**	***n*=**	**p-ERKl/2 (IHC)**	**p-ERKl (WB)**	**p-ERK2 (WB)**	**ERK-I (WB)**	**ERK-2 (WB)**
*(A) P-values after χ*^*2*^ *test*^a^						
Clinical/histological parameters						
Age (20–50 *vs* >50 years)	148	NS	NS	NS	NS	NS
Stage (I *vs* II *vs* III/IV)	140	0.020^*^	NS	0.014^*^	NS	NS
Histol. type (ductal *vs* others)	148	NS	NS	NS	NS	NS
Nodal status (positive *vs* negative)	139	0.003^*^	NS	NS	NS	NS
ER status (IHC; pos. *vs* neg.)	146	NS	NS	NS	**0.007**	NS
Recurrence	120	0.024^*^	0.007^*^	0.062^*^	NS	NS
DOD	120	0.072^*^	0.008^*^	0.063^*^	NS	NS

Proliferation-associated proteins (WB)						
Cyclin Dl	75	0.077^*^	NS	NS	**0.023**	**0.014**
Rb	75	NS	NS	NS	NS	**0.031**
Rb2	75	NS	NS	NS	NS	0.094
p27	68	NS	NS	NS	NS	NS
pl6	75	NS	NS	NS	NS	**0.023**
p2l	75	NS	NS	NS	NS	NS
Cyclin E	75	NS	NS	NS	NS	NS
Ki67	75	NS	NS	NS	NS	NS

Invasion-associated proteins (WB)						
MMP9	74	NS	0.039^*^	NS	NS	NS
MMP1 proenzyme	74	NS	NS	NS	0.090	NS
MMP1 active	74	NS	**0.020**	NS	NS	NS
PAI-1	75	NS	NS	NS	**0.029**	NS

AP-I transcription factors (WB)						
c-Jun	75	NS	NS	NS	NS	NS
JunB	75	NS	NS	NS	NS	NS
JunD	75	NS	0.062^*^	NS	NS	**0.008**
c-Fos	75	NS	NS	NS	NS	**0.038**
FosB	75	NS	0.073	NS	**0.015**	NS
Fra-1	75	NS	NS	NS	NS	NS
Fra-1 (phosph.)	75	NS	0.086^*^	NS	NS	NS
Fra-2	75	NS	NS	NS	**0.035**	**0.048**
Fra-2 (phosph.)	75	NS	NS	NS	NS	NS

High expression of….	…. is significantly associated with:					
*(B) Overview of significant statistical associations* b						
p-ERK (IHC)	Early stage; negative nodal status; low frequency of recurrence					
p-ERK1 (WB)	Low frequency of recurrence and DOD; low MMP9 and high MMPI expression levels					
p-ERK2 (WB)	Early stage					
ERKl (WB)	Positive ER status; high cyclin D1, PAI-1, FosB and Fra-2 expression					
ERK2 (WB)	High cyclin Dl, Rb, p16, JunD, c-Fos and Fra-2 expression					

ER=estrogen receptor; NS=not significant; WB=Western blot results; IHC=immunohistochemical data; DOD=death of disease.

a *P*-values after *χ*^2^ tests below 0.1 are shown. Statistically significant correlations are given in bold letters and inverse correlations are indicated by asterisks. The formation of groups for statistical analysis is described in Materials and Methods.

b Only correlations with *P*<0.05 are included.

**Table 2:** Multivariate analysis for relapse-free and overall survival as a function of p-ERK1/2 expression^a^

**Category**	**Beta (s.e.)**	**Relative risk (Cl)**	***P*-value**
*Relapse-free survival: p-ERK1 (Western blot)*			
Stage			
Stage 1			0.018
Stage 2	−0,105 (0.534)	0.900 (0.32–2.56)	0.843
Stages 3+4	1.406 (0.644)	4.080 (1.15–14.42)	0.029

p-ERK1 (WB)			
Strong			0.049
Moderate	0.871 (0.691)	2.390 (0.62–9.26)	0.208
Weak	1.515 (0.648)	4.551 (1.28–16.21)	0.019

*Relapse-free survival; p-ERK1/2 (immunohistochemistry)*:			
p-ERK1/2 (IHC)			
>20% positive			0.017
1–20% positive	2.111 (0.771)	8.257 (1.82–37.41)	0.007
Negative/focal	2.158 (0.795)	8.656 (1.82–41.15)	0.006

*Overall survival; p-ERK1 (Western blot)*:			
Grading			
G1+2			
G3	0.871 (0.459)	2.389 (0.97–5.87)	0.058
p-ERK1 (WB)			
Strong			0.038
Moderate	0.449 (0.733)	1.57 (0.37–6.59)	0.540
Weak	1.414 (0.646)	4.11 (1.16–14.59)	0.029

*Overall survival; p-ERK1/2 (immunohistochemistry):*			
Grading			
G1+2			
G3	1.170 (0.546)	3.22 (1.11–9.40)	0.032
p-ERK1/2 (IHC)			
>20% positive			0.058
1–20% positive	1.799 (0.786)	6.04 (1.30–28.20)	0.022
Negative/focal	1.864 (0.854)	6.45 (1.21–34.40)	0.029

s.e.=standard error; CI=95% confidence interval; WB=Western blot; IHC=immunohistochemical.

a Cox's regression analysis using forward selection of the variables including stage, grading, ER status and nodal involvement together with either p-ERK1 expression as measured by immunoblots or p-ERK1/2 expression in tumour cells as determined by immunohistochemistry.
